# Catching up with old friends: a 2-year survey of phlebotomine sand fly-borne phleboviruses presence in southern Portugal

**DOI:** 10.1186/s13071-026-07394-1

**Published:** 2026-04-19

**Authors:** Carla Maia, Ricardo Parreira, Elif Kurum, Federico Lucchese, João Cruz, Rafael Marmé, Yasmina Martinez-Barciela, Alejandro Polina, Rémi Charrel, Nazli Ayhan

**Affiliations:** 1 Global Health and Tropical Medicine (GHTM), Associate Laboratory in Translation and Innovation Towards Global Health, LA-REAL, Instituto de Higiene e Medicina Tropical (IHMT), Universidade Nova de Lisboa (UNL), Lisbon, Portugal; 2 Unité des Virus Émergents (UVE: Aix-Marseille UnivUniversità di Corsica, IRD 190, Inserm 1207, IRBA), Marseille, France; 3 Departamento de Ecoloxía e Bioloxía Animal, Universidade de Vigo, Vigo, Spain; 4 National Reference Center for Arboviruses, Inserm-IRBA, Marseille, France

**Keywords:** Alcube virus, Arrabida virus, Massilia virus, Toscana virus, Phlebovirus, Phenuiviridae, Portugal, Sand fly

## Abstract

**Background:**

Phlebotomine sand flies are vectors of several pathogens of medical and veterinary relevance worldwide, including three human viruses of the genus *Phlebovirus* in Europe: sand fly fever Sicilian virus (*Phlebovirus siciliaense*, SFSV), Toscana virus (*Phlebovirus toscanaense*, TOSV), and sand fly fever Naples virus (*Phlebovirus napoliense*, SFNV). Most human infections are asymptomatic or cause influenza-like illness; however, TOSV can cause severe neurological disease. Within the framework of the European Climate Monitoring and Decision Support Framework for Sand Fly–borne Diseases (CLIMOS) project, this study reports the molecular detection of sand fly-borne phleboviruses over 2 years of entomological surveillance in southern Portugal, aiming to characterize their genetic diversity and geographic distribution.

**Methods:**

Phlebotomine sand flies were collected using Centers for Disease and Control (CDC) miniature light traps between April and November of 2023 and 2024 in different regions of southern Portugal, including the Lisbon Metropolitan Area and the Algarve region. Additional collections were carried out in the Portalegre district between June and October 2024. After morphological identification, specimens were pooled according to species, sex, collection date, and location. Engorged females were analyzed individually. Molecular screening included a conventional polymerase chain reaction (PCR) for pan-*Phlebovirus* detection and a real-time reverse transcription quantitative (RT-q)PCR for the specific detection of TOSV and SFSV.

**Results:**

A total of 7719 sand flies were collected—4131 in 2023 (620 pools and 305 engorged females) and 3588 in 2024 (1062 pools and 393 engorged females). *Phlebotomus perniciosus* was the dominant species in both years and across all regions. Other identified species included *Phlebotomus ariasi*, *Phlebotomus sergenti*, *Phlebotomus papatasi*, and *Sergentomyia minuta*. Four *Phlebovirus*-positive pools were identified, all from *P. perniciosus* collected in the Algarve region—two in 2023 and two in 2024, including TOSV (*n* = 1), Alcube virus (*n* = 1), *Phlebovirus* strain PoSFPhlebV/21/2007 related to Massilia virus (*n* = 1), and Arrabida virus (*n* = 1) accordingly partial L segment sequence analysis.

**Conclusions:**

This study highlights the diversity and ongoing co-circulation of sand fly-borne phleboviruses in southern Portugal, emphasizing the need for surveillance and diagnostic efforts to encompass a broader range of *Phlebovirus* species. The consistent association of *P. perniciosus* with multiple viral species and locations reinforces its role as a key vector, warranting targeted vector-management strategies and inclusion in risk-prediction models.

**Graphical abstract:**

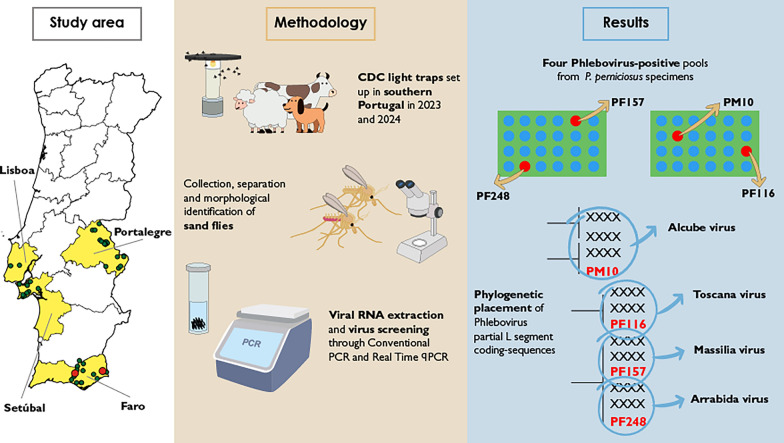

## Background

Phlebotomine sand flies (Diptera: Psychodidae) are vectors of several pathogens of medical and veterinary importance worldwide. Among these, phleboviruses (Order: Hareavirales, Family: Phenuiviridae), are a group of tripartite, single-stranded RNA viruses that include multiple viral species circulating throughout the Mediterranean region [15].

In Europe, sand fly-borne viruses of the *Phlebovirus* genus include three human pathogens, sand fly fever Sicilian virus (*Phlebovirus siciliaense* or SFSV), Toscana virus (*Phlebovirus toscanaense* TOSV) and sand fly fever Naples virus (*Phlebovirus napoliense*, SFNV) [1]. Proven and suspected vectors within these complexes include several *Phlebotomus* species, such as *Phlebotomus perniciosus*, *Phlebotomus perfiliewi* for TOSV, and *Phlebotomus papatasi* for SFSV [2, 3]. Although most human infections are asymptomatic or cause mild, influenza-like illness, some phlebovirus infections can lead to severe neurological disease. Notably, TOSV is one of the recognized causes of meningitis and meningoencephalitis during the warm months in endemic areas of the Mediterranean basin [4], while SFSV and SFNV have historically been associated with “flu-like” signs and symptoms [2].

Portugal was the second country to be recognized as endemic for TOSV, following the report of neuroinvasive disease in a Swedish tourist returning from the Algarve, in the south of the country, in the early 1980s [5]. Subsequent case reports and retrospective studies have confirmed additional acute TOSV infections, as well as one acute SFSV infection in both residents and travelers exposed in Portugal [4, 6, 7].

In recent years, research efforts aimed at mapping the diversity, distribution, and public and animal health impact of sand fly-borne pathogens in Portugal has been intensified. Serological surveys have demonstrated the presence of neutralizing antibodies against TOSV and SFSV in humans from southwest Portugal [8] and in blood donors from mainland regions [9, 10], confirming widespread, circulation of both viruses. High seroprevalence rates for SFSV (up to 56.3%) and lower rates for TOSV (up to 6.8%) have been found in dogs from southern Portugal [11, 12]. In cats, reported seroprevalence is lower, with values up to 4.9% for TOSV and 2.2% for SFSV [11, 13]. Exposure to TOSV has also been documented in wild animals, including foxes and wolves in Portugal [14].

Five sand fly species are endemic to Portugal: *P. perniciosus*, *Phlebotomus ariasi*, *Phlebotomus sergenti*, *P. papatasi*, and *Sergentomyia minuta*, while recent studies have suggested the involvement of a broader range of sand fly species as potential vectors of TOSV [15]. To date, Alcube, Arrabida, Massilia, and Toscana phleboviruses have been isolated or molecularly detected in sand flies collected in the south of Portugal [16, 17].

Within the framework of the European Climate Monitoring and Decision Support Framework for Sand Fly–borne Diseases (CLIMOS) project, aimed at developing an early warning system to predict changes in the distribution and seasonality of sand fly vector species and to assess the risk of sand fly-borne infections across southern European countries, Turkey, and Israel, this study focuses on southern Portugal. It reports the molecular detection of phleboviruses transmitted by sand flies collected during entomological surveillance conducted in 2023 and 2024 with the aim of characterizing their diversity and geographic distribution in the country.

## Methods

### Field collection and processing of adult phlebotomine sand fly specimens

Phlebotomine sand flies were collected throughout the activity season, between April and November of 2023 and 2024, in different regions of southern Portugal: the Lisbon Metropolitan Area (LMA) and the Algarve region. Field collections were also performed in Portalegre district between June and October 2024. Sampling collection followed the standardized protocol established within the framework of the CLIMOS project using CDC miniature light traps (without lures or CO_2_ supplementation) in areas where animal hosts were present, such as livestock farms and kennels, both indoors and outdoors. In most of the biotopes studied, the main vertebrate hosts available within a 50 m radius were goats, sheep, cattle, dogs, cats, horses, and birds (poultry and exotic species).

CDC traps were set up before sunset and collected the next day after sunrise and collected insects were transported from the sampling sites to the laboratory in cool boxes. Sand flies were morphologically identified to species level by dissecting their three terminal abdominal segments on a cold surface (e.g., a Petri dish on ice), which were slide-mounted and cleared using Marc-André solution [18]. The preparation of monospecific pools was defined according to the sex and total number of individuals captured. Every specimen from low-yield collections (less than 200 individuals) was identified morphologically, whereas for captures ranging between 200 and 1000 individuals, morphological identification was performed on the first 200 specimens plus 10% of all additional specimens comprising equal numbers of females and males (50% each). Following morphological identification, sand flies were pooled (maximum 30 individuals per tube) according to species, sex, collection date, and location, and stored at −80 °C until further analysis. Engorged females were analyzed individually to enable future identification of blood meal source.

### RNA extraction

Specimen mechanical homogenization was performed in 700 μL of minimum essential medium (MEM) solution by vortexing with 0.2 mm tungsten beads. To remove solid debris, homogenates were centrifuged at 8000 rpm for 5 min at 4 °C. Each homogenate was subsequently divided: 100 μL for biobanking and virus isolation, and 200 μL for total nucleic acid extraction using the QIAamp Viral RNA Mini Kit (Qiagen, USA). The remainder homogenate was kept in the original tube, and together with the biobanking aliquot, were stored at −80 °C until further analysis (Table 1).

**Table 1:** Numbers of sand flies analyzed in the course of this work. Specimens were collected in 2023 and 2024, were separated by species and sex, and analyzed either in pools (up to 30 specimens) or individually (engorged females)

Region	Number of pools M	Number of pools F	Total pools	% Pools M	% Pools F	Blood fed females	% Total fed
2023							
LMA	70	27	97	72.16%	27.84%	41	13.44%
Algarve	254	269	523	48.57%	51.43%	264	86.56%
Total	324	296	620	52.26%	47.74%	305	
2024							
LMA	44	46	90	48.89%	51.11%	13	3.31%
Algarve	184	456	640	28.75%	71.25%	266	67.68%
Portalegre	114	218	332	34.34%	65.66%	114	29.01%
Total	342	720	1062	32.20%	67.80%	393	

*F* female specimens, *M* male specimens, *LMA* Lisbon Metropolitan Area

### Viral sequence detection

Conventional RT-PCR for pan-*Phlebovirus* detection (referred to as “Pan-Phlebovirus” in Table 2) was performed essentially as described by Matsuno et al. [19]. This assay targets a 514 bp region of the L segment encoding the RNA-dependent RNA polymerase (RdRp) gene [19], using a mixture of the TBPVL2759FppL1, TBPVL3267RppL2, HRT-GL2759F, and HRT-GL3276R primers in each amplification reaction.

**Table 2:** Summary of *Phlebovirus* detection results from sand fly pools of all samples collected in Portugal. Each row indicates the sample ID, conventional RT-PCR (pan-phlebovirus), and realtime RT-qPCR (TOSV-Trio and pan-SFSV) results, sequence identification result, and corresponding GenBank accession number. Nucleotide acid sequence similarities were calculated on the basis of the partial L segment encoding the RNA-dependent RNA polymerase sequence (RdRp)

Pool code	Place of collection/region_/_year	Vector species	Sand flies per pool	Segment	bp	% NAI	Closely related virus
PF157	Portela da Nave/Algarve/2023	*P. perniciosus*	22 females	L	503	96.95	*Phlebovirus* strain PoSFPhlebV/21/2007 related to Massilia virus
PF248	Cerro do Enho/Algarve/2023	*P. perniciosus*	30 females	L	484	96.70	Arrabida virus
PF116	Portela da Nave/Algarve/2024	*P. perniciosus*	30 females	L	428	97.66	Toscana virus isolate Spain
PM10	Cerro do Enho/Algarve/2024	*P. perniciosus*	4 males	L	479	94.15	Alcube virus strain S20

P. indicates *Phlebotomus*; *bp* base pair, *NAI* nucleic acid identity

SuperScript™ III One-Step RT-PCR System with Platinum™ Taq DNA Polymerase (Thermo Fisher Scientific, USA) [20] was utilized. Further, real-time RT-qPCR amplification of TOSV [21–23] and SFSV [11], referred to as TOSV-Trio and pan-SFSV in Table 2, respectively, were performed using SuperScript III Platinum One-Step qRT-PCR System (Thermo Fisher Scientific, USA). Amplicons obtained by conventional PCR were purified by NucleoSpin® Gel and PCR Clean-up kit NucleoSpin Gel and PCR Clean up kit (Macherey Nagel, Germany) and Sanger sequenced. For TOSV/SFSV real-time RT-qPCR assays, negative amplification results were considered those with a cycle threshold (Ct) above 35.

The sequences described in this report have been assigned to the Genbank® with the accession numbers LC911388–LC911391.

### Virus isolation

For each sand fly sample that tested positive by pan-*Phlebovirus* RT-PCR or real time RT-qPCR assay, 50 µL of homogenized material was mixed with 350 µL of MEM containing 1% L-glutamine, nonessential amino acids, penicillin–streptomycin (200 mM), and 3% amphotericin B. The mixture was inoculated onto Vero E6 cell monolayers seeded in six-well plates and incubated for 1 h at 37 °C in a humidified atmosphere with 5% CO_2_. After incubation, the inoculum was transferred to a second well, and 2.5 mL of enriched MEM supplemented with 5% fetal bovine serum (FBS). Cell cultures were monitored daily for the presence of cytopathic effects (CPE) and subjected to two consecutive passages. At each passage, 200 µL of culture supernatants were harvested and analyzed using a conventional pan-*Phlebovirus* RT-PCR assay.

### Viruses minimum infection rate estimation

Following the molecular detection analysis, the minimum infection rate (MIR) was calculated under the assumption that only one individual was infected within each positive pool, using the formula: (number of positive pools/total number of specimens tested) × 1000 [24].

### Phylogenetic analysis

To assess the phylogenetic relationships of the *Phlebovirus* sequences obtained in the course of this study with other members of the genus, they were included in a dataset comprising a total of 57 homologous *Phlebovirus* references downloaded from the NCBI Virus database (https://www.ncbi.nlm.nih.gov/labs/virus/vssi/#/). These references represented recognized and/or proposed species or species complexes. Sequences displaying stretches of “N” or smaller than 400 nucleotides were removed from the dataset.

Nucleotide sequences were first aligned with MAFFT v7 [25] and then manually curated to ensure codon-by-codon alignment using Aliview [26]. Before phylogenetic analysis was executed, a preliminary evaluation of the dataset phylogenetic signal was carried out using likelihood mapping in TREE-PUZZLE v5.3 [27]. Maximum likelihood phylogenetic reconstruction was run on an Ubuntu server using the IQ-TREE v2.4.0 software [28], applying the evolutionary model (GTR + F + I + G4) previously identified by the same software under all available criteria (Akaike information criterion (AIC), corrected AIC, and Bayesian information criterion). The obtained phylogenetic tree was visualized and edited in FigTree v1.3.1 (https://tree.bio.ed.ac.uk/software/figtree/).

## Results

A total of 7719 sand flies were collected and tested: 4131 in 2023 (620 pools and 305 engorged females) and 3588 in 2024 (1062 pools and 393 engorged females) (Table 1). *Phlebotomus perniciosus* was the dominant species in 2023 in the Algarve (78.2%, *n* = 2820) and LMA (88.3%, *n* = 455), with lesser occurrences of *P. sergenti*, *P. ariasi*, and *S. minuta*; sand fly activity in the Algarve began in April and continued through November (peaks in June and September), while in the LMA it spanned April–November (peaks in June and August). In 2024, *P. perniciosus* remained dominant in the three sampling regions: Algarve (69.0%, *n* = 1715), LMA (84.6%, *n* = 159), and Portalegre (61.0%, *n* = 532), with lesser occurrences of *P. sergenti*, *P. ariasi*, and *S. minuta*. *Phlebotomus papatasi* was also recorded in the Algarve (17 specimens; 6 in 2023 and 11 in 2024). In 2024, sand fly activity in the Algarve began in April and persisted through November (peaks in June and August), in the LMA from May to November (peaks in June and September), and in Portalegre from June to September (peak in August).

**Fig. 1:**
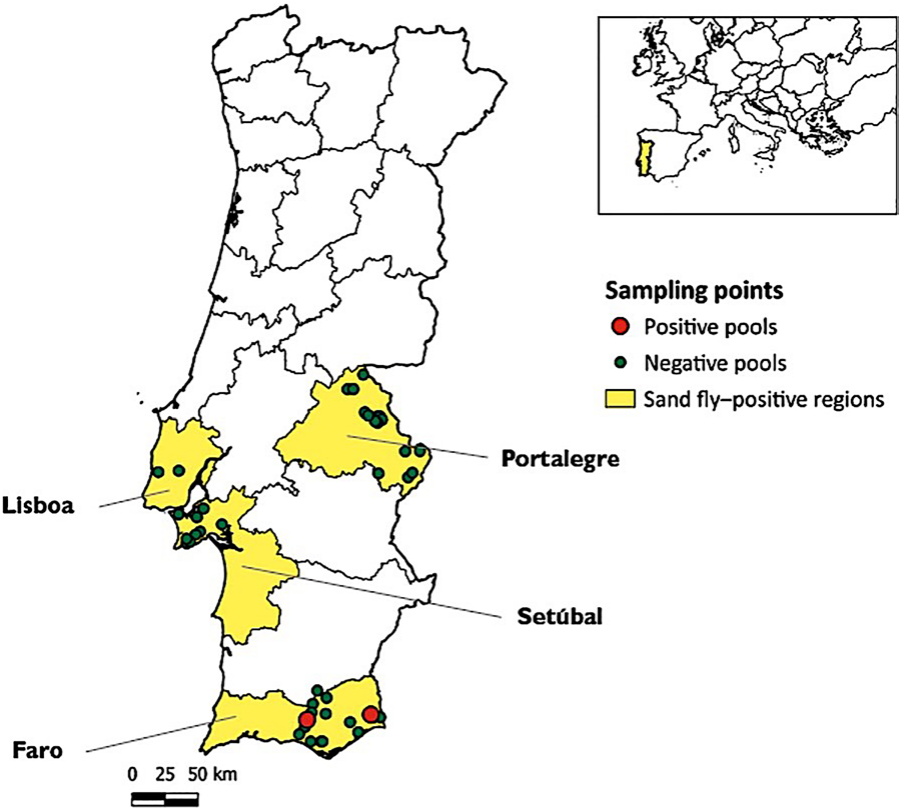
Map of Portugal showing sampled regions and locations of positive pools

A total of four *Phlebovirus-*positive pools were identified using the pan-*Phlebovirus* RT-PCR assay, all from *P. perniciosus* specimens collected in the Algarve region (Fig. 1). Two positive pools were identified in 2023 (pool PF157 amplified from a pool of females collected in May and pool PF248 amplified from a pool of females collected in June) and two from 2024 (pool PM10 amplified from a pool of males collected in May and pool PF116 amplified from a pool of females collected in August). All engorged females tested negative by conventional RT-PCR and real-time RT-qPCR assays. When considering the total number of *Phlebovirus*-positive amplification results, the calculated MIR for each collection year (Table 2) was 0.52 (2/3871) and 0.73 (2/2751) when considering the total number of specimens collected in 2023 and 2024, respectively. However, since the combined Basic Local Alignment Search Tool (BLAST)/phylogenetic analyses results indicated the detection of a genetically different virus in each of the four positive *Phlebovirus* pools, the MIR (per each virus) totaled 0.13 (1/7719).

Although all 1684 pools and 698 engorged females were tested using SFSV- and TOSV-specific qRT-PCR assays, only pool PF116 was TOSV positive. Attempts to isolate virus from the phlebovirus positive samples were unsuccessful.

**Fig. 2:**
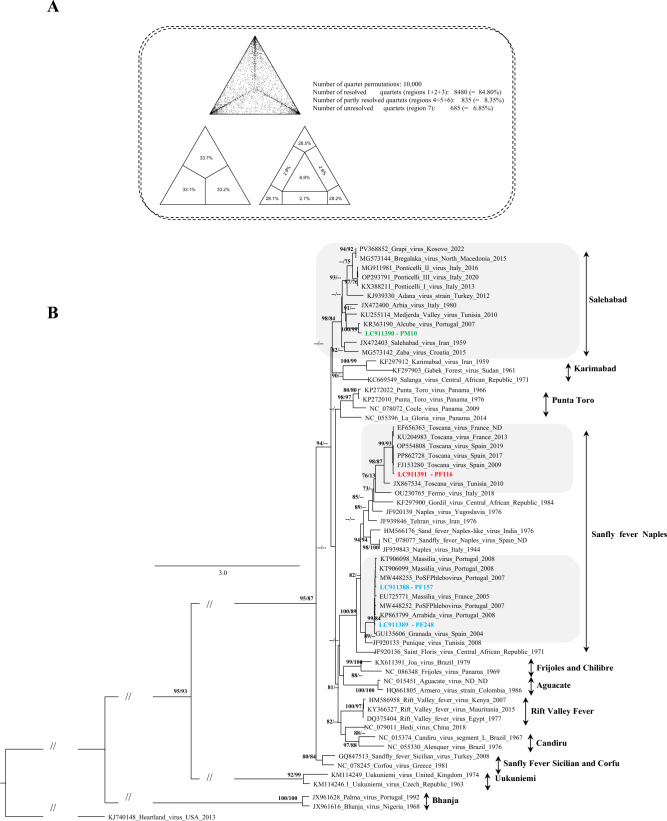
Phylogenetic signal (**A**) and tree (**B**) analyses of partial *Phlebovirus* L sequences. At the main tree branches approximate likelihood ratio (aLRT)/conventional bootstrap values > 75% are indicated. Taxon information includes the GenBank accession number_virus name_geographic origin_sampling date. Recognized and/or proposed *Phlebovirus* species or species complexes are noted to the right of the trees. The viral sequences obtained in this study are highlighted in color inside the shaded boxes (indicated by their accession number and sample ID). The scale bar denotes nucleotide substitutions per site. Evaluation of the dataset phylogenetic signal was carried out using likelihood mapping in TREE-PUZZLE v5.3, and Maximum likelihood phylogenetic reconstruction was performed using IQ-TREE v2.4.0 on an Ubuntu server

Phylogenetic placement of the *Phlebovirus* partial L segment coding-sequences into previously characterized monophyletic clusters was assessed on the basis of the maximum likelihood optimization criterion under the GTR + F + I + G4 evolutionary model, using a dataset of a total of 61 sequences characterized by a high phylogenetic signal (84.8% of totally resolved sequence quartets after 10,000 resamplings; Fig. 2A as defined by likelihood mapping). Genome analysis of partial L segment sequences showed that the sample PM10 (Genbank® accession number LC911390) was most closely related to Alcube virus (ACBV; *Phlebovirus alcubeense*), while sample PF157 partial L segment sequence (accession number LC911388) showed high nucleic acid sequence similarity to *Phlebovirus* strain PoSFPhlebV/21/2007 related to Massilia virus (Table 2). For the two remaining positive pools, sample PF248 (accession number LC911389) was most closely related to Arrabida virus (*Phlebovirus napoliense*) and sample PF116 (accession number LC911391) was most closely related to TOSV isolate Spain (Table 2).

## Discussion

Phleboviruses transmitted by sand flies are endemic and emerging pathogens in the Mediterranean, with TOSV and SFSV posing the greatest risk in the Iberian Peninsula. The epidemiological landscape concerning the circulation of sand fly-borne phleboviruses in Portugal has been previously characterized and involves the co-circulation of at least five viral species: TOSV, SFSV, Alcube, Arrabida, and Massilia viruses [16]. Most of them represent a neglected public health threat that often manifests as undifferentiated summer fevers or neuroinvasive diseases [2, 3, 16, 17].

TOSV is the most clinically significant sand fly-borne phlebovirus in Portugal due to its neurotropism [29]. Recent nationwide studies focused on blood donors estimated a true human seroprevalence of 2.6%, with regional hotspots detected in the Alto Alentejo (inland, central–southern half of the country) and Douro (inland, mountainous, river‑valley region, in the north) regions, where TOSV-seroprevalence values were 14.8% and 13.3%, respectively [10]. This virus has been linked to sporadic cases of aseptic meningitis and encephalitis, with lineage B being the predominant circulating genotype in Portugal [4, 17]. While antibodies have long been detected in humans and domestic and wild animals [7, 10–14] the first detection in Portugal of TOSV in its proven vector *P. perniciosus* occurred only recently, in 2023, when viral RNA was identified in sand flies from the southern region of the country [17]. This likely reflects the low priority given to these viruses by public health authorities until 2016, when sand flies were included in Portugal’s National Vector Surveillance Network (REVIVE program) [17], with prior monitoring efforts being limited, fragmented, and mainly dependent on research projects.

Further, SFSV, which typically causes self-limiting febrile syndrome, appears to be more geographically widespread when compared with TOSV [9]. A nationwide study estimated a human seroprevalence of 4.7%, with regional positivity reaching 11.9% in areas such as the LMA and Alentejo (southern Portugal) [9]. In animal populations, SFSV shows remarkably high exposure rates. Indeed, a study in sheltered dogs from southern Portugal revealed a seroprevalence of 56.3% [12], but despite this strong serological evidence, no SFSV strain has yet been isolated from either Portuguese sand flies or vertebrates’ hosts [9].

Other phleboviruses, such as Massilia, Arrabida, and Alcube viruses, have been isolated during entomological surveys in southern Portugal [30]. Massilia virus is widely distributed in the Setúbal district (just south of Lisbon, on the south bank of the Tagus estuary and part of the LMA) and Faro district (Algarve region), where two genomic variants co-circulate [30]. Alcube virus, discovered in the Arrábida region (in the Setúbal district), remains a species of undetermined pathogenic potential, with Setúbal and Algarve regions being considered biodiversity hotspots for phleboviruses due to the high diversity of viral signatures detected there [30]. Further, Arrabida virus had been detected in sand flies in the Arrábida region, which is closely related to Granada virus, identified in Spain [31]. Our findings are consistent with those of Remoli et al. [32], who reported the detection of TOSV and Arrabida virus in *P. perniciosus* in southwestern Madrid, Spain, suggesting possible regional circulation of related *Phlebovirus* strains [32].

The phylogenetic analysis of the L‑segment sequences obtained during this 2‑year survey supports the notion that southern Portugal harbors a diverse community of sand fly‑borne phleboviruses. Phylogenetic reconstructions using a dataset of nucleotide sequences with a high phylogenetic signal disclosed the dispersal of four newly generated sequences (Fig. 1), and underscored either repeated introductions or long‑standing co‑circulation of several phlebovirus species in Portugal, rather than local expansion of a single viral strain.

The detection of PM10 sample clusters with Alcube virus detected in a pool of *P. perniciosus* males from the Algarve (Cerro do Enho) suggest that viruses typically associated with more eastern or extra‑Iberian settings may be present, or at least transiently introduced, in the south of Portugal. Further, the fact that this signal was obtained from a male pool argues against passive detection of viral RNA through blood feeding, being more consistent with true infection, potential vector competence, and/or the possibility of vertical or transovarial maintenance of these viruses in the vector population. While the presence of Alcube virus in Portugal had already been described in the LMA (Setúbal district) [33], this study reports its first detection of the virus in the Algarve region. These findings raise questions regarding their ecological niche, reservoir range, and possible, albeit currently undocumented, clinical impact in humans or other mammals.

In addition, sample PF116 clusters firmly with TOSV reference sequences. The detection of TOSV RNA in *P. perniciosus* females at Portela da Nave in late summer 2024 is consistent with previous entomological and clinical reports, linking this vector–virus pairing to human neuroinvasive disease in Portugal [5, 6, 32], and it corroborates the role of the Algarve as an area where humans, sand flies, and TOSV coexist during peak transmission season. In the context of growing evidence of TOSV circulation and imported neuroinvasive cases, these data underscore the need to systematically include TOSV in the differential diagnosis of aseptic meningitis and encephalitis in southern Portugal.

The remaining two samples PF157 and PF248 cluster with *Phlebovirus* strain PoSFPhlebV/21/2007 (closely related with Massilia virus) and Arrabida viruses, both detected in *P. perniciosus* pools from the Algarve. This sublineage has been repeatedly reported across the western Mediterranean basin and already includes phlebovirus sequences previously obtained in southern Portugal, particularly from Arrábida and other sites [16, 29, 31, 33], which are now joined by the Portela da Nave and Cerro do Enho strains. The recurrence of closely related Massilia/Arrabida/Granada‑like viruses over multiple years and different geographic locations suggests that these viruses are well established in local sand fly populations rather than representing sporadic introductions. Once again, whether these newly identified phleboviruses cause mild, underdiagnosed febrile disease or are largely nonpathogenic in humans remains to be determined. Regardless, their repeated detection in southern Portugal argues for continued ecological and clinical surveillance.

Despite the observed genetic diversity among the sand fly-borne phleboviruses, overall *Phlebovirus* detection was similar between years, with a MIR of 0.52 in 2023 and 0.73 in 2024, reflecting a low but consistent level of virus circulation. However, because combined BLAST/phylogenetic analyses revealed that each of the four positive *Phlebovirus* pools contained a genetically distinct virus variant, the MIR calculated per individual virus was 0.13 (1 virus infection per 7719 sand flies). This low per-virus MIR is comparable to the recently reported detection of TOSV in wild-caught sand flies in Portugal, where one positive pool of 30 females out of 642 flies tested in 2023 yielded a MIR of 0.16 [17].

This study underscores the widespread diversity and ongoing circulation of sand fly-borne phleboviruses in Portugal, with a focus on the southern regions. The confirmed presence of TOSV, Alcube virus, Massilia virus, and Arrabida virus indicates their sustained presence and extensive geographic distribution within the country. Our findings further emphasize that diagnostic and surveillance algorithms for sand fly‑borne viruses in southern Portugal must look beyond TOSV alone and should consider a broader panel of phleboviruses. In parallel, the repeated implication of *P. perniciosus* as the vector for these viruses across different viral species and geographic localities highlights its central role as the main phleboviruses bridge vector in Portuguese-Mediterranean‑type ecosystems, establishing it as a primary focus for both vector-management strategies and the development of risk-prediction models [34, 35].

## Conclusions

This study reinforces the circulation of diverse sand fly-borne phleboviruses in Portugal. Continued vector surveillance, improved diagnostics, and assessment of their clinical impact are needed to clarify transmission dynamics, monitor diversity, track emerging strains, and evaluate public and animal health risks.

## Data Availability

The data supporting the conclusions of this article are included within the article.

## Declarations

### Generative AI and AI-assisted technologies in the writing process

During the preparation of this work the authors used Perplexity AI tool to improve the readability and language of the manuscript. After using this tool, the authors reviewed and edited the content as needed and take full responsibility for the content of the published article.

## References

[1] Calisher CH, Calzolari M. Taxonomy of Phleboviruses, emphasizing those that are sandfly-borne. Viruses. 2021;13:918. 10.3390/v13050918. 34063467 10.3390/v13050918 PMC8156068

[2] Alkan C, Bichaud L, De Lamballerie X, Alten B, Gould EA, Charrel RN. Sandfly-borne phleboviruses of Eurasia and Africa: epidemiology, genetic diversity, geographic range, control measures. Antivir Res. 2013;100:54–74. 10.1016/j.antiviral.2013.07.005. 23872312 10.1016/j.antiviral.2013.07.005

[3] Ayhan N, Prudhomme J, Laroche L, Bañuls A-L, Charrel RN. Broader geographical distribution of Toscana virus in the Mediterranean region suggests the existence of larger varieties of sand fly vectors. Microorganisms. 2020;8:114. 10.3390/microorganisms8010114. 31947561 10.3390/microorganisms8010114 PMC7022675

[4] Ayhan N, Eldin C, Charrel R. Toscana virus: a comprehensive review of 1381 cases showing an emerging threat in the Mediterranean regions. J Infect. 2025;90:106415. 10.1016/j.jinf.2025.106415. 39828129 10.1016/j.jinf.2025.106415

[5] Ehrnst A, Peters CJ, Niklasson B, Svedmyr A, Holmgren B. Neurovirulent Toscana virus (a sand fly fever virus) in Swedish man after visit to Portugal. Lancet. 1985;1:1212–3. 10.1016/s0140-6736(85)92886-7. 2860406 10.1016/s0140-6736(85)92886-7

[6] Santos L, Simões J, Costa R, Martins S, Lecour H. Toscana virus meningitis in Portugal, 2002-2005. Euro Surveill. 2007;12:3–4. 10.2807/esm.12.06.00715-en. 10.2807/esm.12.06.00715-en 17991401

[7] Amaro F, Luz T, Parreira P, Ciufolini MG, Marchi A, Janeiro N, Zagalo A, Proença P, Ramos MI, Alves MJ. Toscan a virus in the Portuguese population: serosurvey and clinical cases. Acta Med Port. 2011;24(Suppl 2):503-8. 22849940

[8] Maia C, Ayhan N, Cristóvão JM, Pereira A, Charrel R. Human seroprevalence of Toscana virus and Sicilian phlebovirus in the southwest of Portugal. Eur J Clin Microbiol Infect Dis. 2022;41:137–41. 10.1007/s10096-021-04332-0. 34389911 10.1007/s10096-021-04332-0

[9] Rocha R, Kurum E, Ayhan N, Charrel R, Maia C. Seroprevalence of sand fly fever Sicilian virus in blood donors in mainland Portugal. Parasit Vectors. 2025;18:261. 10.1186/s13071-025-06885-x. 40618143 10.1186/s13071-025-06885-x PMC12228258

[10] Rocha R, Kurum E, Ayhan N, Charrel R, Maia C. Seroprevalence of Toscana virus in blood donors in mainland Portugal. Parasit Vectors. 2025;18:82. 10.1186/s13071-025-06726-x. 40033431 10.1186/s13071-025-06726-x PMC11874794

[11] Alwassouf S, Maia C, Ayhan N, Coimbra M, Cristovao JM, Richet H, Bichaud L, Campino L, Charrel RN. Neutralization-based seroprevalence of Toscana virus and sandfly fever Sicilian virus in dogs and cats from Portugal. J Gen Virol. 2016;97(11):2816–23. 10.1099/jgv.0.000592. 27589865 10.1099/jgv.0.000592

[12] Maia C, Alwassouf S, Cristóvão JM, Ayhan N, Pereira A, Charrel RN, Campino L. Serological association between Leishmania infantum and sand fly fever Sicilian (but not Toscana) virus in sheltered dogs from southern Portugal. Parasit Vectors. 2017;10(2):92. 10.1186/s13071-017-2023-x 28285587 10.1186/s13071-017-2023-x PMC5346850

[13] Pereira A, Ayhan N, Cristóvão JM, Vilhena H, Martins Â, Cachola P, Henriques J, Coimbra M, Catarino A, Lestinova T,Spitzova T, Volf P, Campino L, Charrel R, Maia C. Antibody response to Toscana virus and sandfly fever Sicilian virus in cats naturally exposed to phlebotomine sand fly bites in Portugal. Microorganisms. 2019;7(9):339. 10.3390/microorganisms7090339. 31514266 10.3390/microorganisms7090339 PMC6780191

[14] Amaro F. Phlebovirus: importancia em Saude Publica em Portugal [PhD Thesis Biology (Microbiology)]. [Lisbon]: Universidade de Lisboa, Faculdade de Ciências; 2010.

[15] Ayhan N, Charrel RN. Sandfly-borne viruses of demonstrated/relevant medical importance. In: Vectors and Vector-Borne Zoonotic Diseases. Intech Open; 2019. 10.5772/intechopen.81023

[16] Amaro F, Zé-Zé L, Alves MJ. Sandfly-borne phleboviruses in Portugal: four and still counting. Viruses. 2022;14:1768. 10.3390/v14081768. 36016390 10.3390/v14081768 PMC9413822

[17] Amaro F, Zé-Zé L, Osório HC, Soares P, Silva M, Freitas IC, Alves MJ. Toscana virus in wild-caught sand flies in Portugal, findings from the National Vector Surveillance Network, 2023. Pathogens. 2024;13(10):905. 10.3390/pathogens13100905. 39452776 10.3390/pathogens13100905 PMC11510618

[18] Pires CA. Contribuição ao conhecimento da distribuição e bioecologia dos flebótomos em Portugal (Diptera, Psychodidae). Bolm Soc Port Ciênc Nat. 1979;197–210.

[19] Matsuno K, Weisend C, Kajihara M, Matysiak C, Williamson BN, Simuunza M, Mweene AS, Takada A, Tesh RB,Ebihara H. Comprehensive molecular detection of tick-borne Phleboviruses leads to the retrospective identification of taxonomically unassigned bunyaviruses and the discovery of a novel member of the genus Phlebovirus. J Virol. 2015;89(1):594-604. 10.1128/JVI.02704-14. 25339769 10.1128/JVI.02704-14 PMC4301164

[20] Ayhan N, Baronti C, Thrion L, Bongiorno G, Maia C, Charrel RN, CLIMOS WP2 Phlebovirus Working Group. External quality assessment for molecular detection of sand fly-borne phleboviruses circulating in the Mediterranean Basin. Parasit Vectors. 2025;18:173. 10.1186/s13071-025-06785-0. 40355941 10.1186/s13071-025-06785-0 PMC12070498

[21] Weidmann M, Sanchez-Seco MP, Sall A, Ly PO, Thiongane Y, Lô MM, Schley H, Hufert FT. Rapid detection of important human pathogenic Phleboviruses. J Clin Virol. 2008;41:138–42. 10.1016/j.jcv.2007.10.001. 18006376 10.1016/j.jcv.2007.10.001

[22] Brisbarre N, Plumet S, Cotteaux-Lautard C, Emonet SF, Pages F, Leparc-Goffart I. A rapid and specific real time RT-PCR assay for diagnosis of Toscana virus infection. J Clin Virol. 2015;66:107–11. 10.1016/j.jcv.2015.03.007. 25866349 10.1016/j.jcv.2015.03.007

[23] Pérez-Ruiz M, Collao X, Navarro-Marí JM, Tenorio A. Reversetranscription, real-time PCR assay for detection of Toscana virus. J Clin Virol. 2007;39:276–81. 10.1016/j.jcv.2007.05.003. 17584525 10.1016/j.jcv.2007.05.003

[24] Gu W, Lampman R, Novak RJ. Assessment of arbovirus vector infection rates using variable size pooling. Med Vet Entomol. 2004;18:200–4. 15189246 10.1111/j.0269-283X.2004.00482.x

[25] Katoh K, Standley DM. MAFFT multiple sequence alignment software version 7: improvements in performance and usability. Mol Biol Evol. 2013;30:772–80. 10.1093/molbev/mst010. 23329690 10.1093/molbev/mst010 PMC3603318

[26] Larsson A. AliView: a fast and lightweight alignment viewer and editor for large datasets. Bioinformatics. 2014;30:3276–8. 10.1093/bioinformatics/btu531. 25095880 10.1093/bioinformatics/btu531 PMC4221126

[27] Schmidt HA, Strimmer K, Vingron M, Von Haeseler A. TREE-PUZZLE: maximum likelihood phylogenetic analysis using quartets and parallel computing. Bioinformatics. 2002;18:502–4. 10.1093/bioinformatics/18.3.502. 11934758 10.1093/bioinformatics/18.3.502

[28] Minh BQ, Schmidt HA, Chernomor O, Schrempf D, Woodhams MD, Von Haeseler A, Lanfear R. IQ-TREE 2: new models and efficient methods for phylogenetic inference in the genomic era. Mol Biol Evol. 2020;37:1530–4. 10.1093/molbev/msaa015. 32011700 10.1093/molbev/msaa015 PMC7182206

[29] Keskek Turk Y, Ergunay K, Kohl A, Hughes J, McKimmie CS. Toscana virus—An emerging Mediterranean arbovirus transmitted by sand flies. J General Virol. 2024;105. 10.1099/jgv.0.002045 10.1099/jgv.0.002045 PMC11542635 39508743

[30] Amaro F, Zé-Zé L, Lourenço J, Giovanetti M, Becker SC, Alves MJ. Phylogenetic analysis of Massilia phlebovirus in Portugal. Viruses. 2021;13:1412. 10.3390/v13071412. 34372617 10.3390/v13071412 PMC8310352

[31] Collao X, Palacios G, De Ory F, Sanbonmatsu S, Pérez-Ruiz M, Navarro JM, Molina R, Hutchison SK, Lipkin WI,Tenorio A, Sánchez-Seco MP. Granada Virus: a natural phlebovirus reassortant of the sandfly fever Naplesserocomplex with low seroprevalence in humans. Am J Trop Med Hyg. 2010;83(4):760-5. 10.4269/ajtmh.2010.09-0697. 10.4269/ajtmh.2010.09-0697 PMC2946739 20889862

[32] Remoli ME, Jiménez M, Fortuna C, Benedetti E, Marchi A, Genovese D, Gramiccia M, Molina R, Ciufolini MG. Phleboviruses detection in *Phlebotomus perniciosus* from a human leishmaniasis focus in South-West Madrid region, Spain. Parasit Vectors. 2016;9:205. 10.1186/s13071-016-1488-3. 27075742 10.1186/s13071-016-1488-3 PMC4831143

[33] Amaro F, Zé-Zé L, Alves MJ, Börstler J, Clos J, Lorenzen S, Becker SC, Schmidt-Chanasit J, Cadar D. Co-circulation of a novel phlebovirus and Massilia virus in sandflies, Portugal. Virol J. 2015;12:174. 10.1186/s12985-015-0407-0. 26497645 10.1186/s12985-015-0407-0 PMC4619550

[34] Amaro F, Luz T, Parreira P, Marchi A, Ciufolini MG, Alves MJ. Serological evidence of Toscana virus infection in Portuguese patients. Epidemiol Infect. 2012;140:1147–50. 10.1017/S0950268811001403. 21798106 10.1017/S0950268811001403

[35] Amaro F, Hanke D, Zé-Zé L, Alves MJ, Becker SC, Höper D. Genetic characterization of Arrabida virus, a novel phlebovirus isolated in South Portugal. Virus Res. 2016;214:19–25. 10.1016/j.virusres.2016.01.004. 26795868 10.1016/j.virusres.2016.01.004

